# Supplementary material for: Identification of photoperiod-induced specific miRNAs in the adrenal glands of Sunite sheep (Ovis aries)
Source: Front Vet Sci. 2022 Jul 22;9:888207. doi: 10.3389/fvets.2022.888207 (PMC9354845; doi:10.3389/fvets.2022.888207)
Supplement: Supplementary file 1 [file Data_Sheet_1.ZIP › Supplementary file/miRDeep2 file.zip!/miRDeep2 file/Know_Structure/SPLP42A3/oar-mir-181a-1.pdf]

miRBase precursor : oar-mir-181a-1
Total read count : 15677
oar-miR-181a read count : 15626
remaining reads : 51

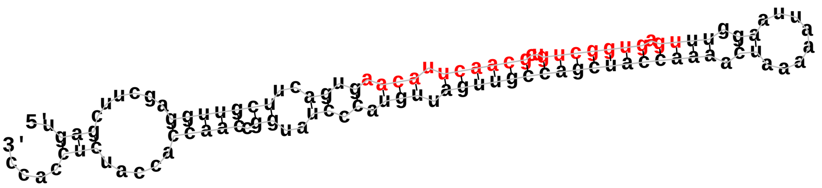

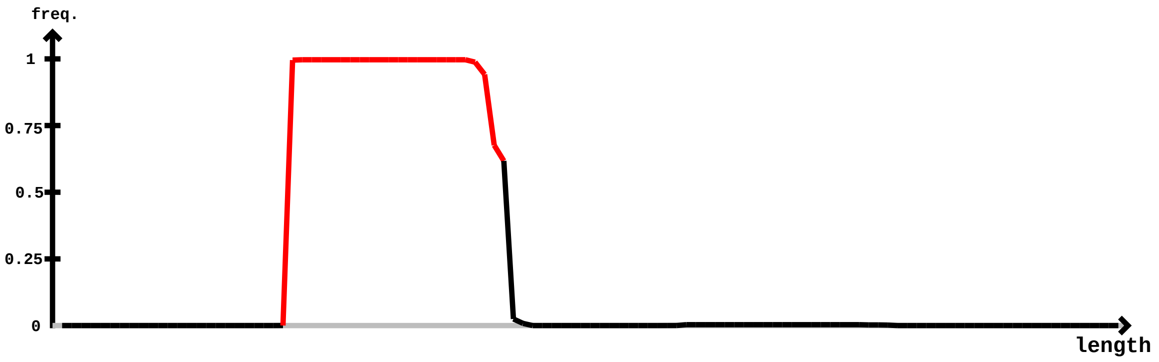


```
                      oar-miR-181a
5'- ugagcuucgagguugcuucagugaacauucaacgcugucggugaguuuggaauuaaaaaucaaaaccaucgaccguugauuguacccuauggccaaccaccaucuccacc   -3'    exp
    .(((......((((((..((.(.(((.((((((..(((((((.((((.((........)).)))))))))))))))))).))).).))..)).))))).....)))....   reads     mm        sample
    ......................gaacauucaacgcugucgguga..................................................................   3         0         seq
    .......................aacauucaacgcugucggu....................................................................   140       0         seq
    .......................aacauucaacgcugucggug...................................................................   721       0         seq
    .......................aacauucaacgcugucgguga..................................................................   4156      0         seq
    .......................aacauucaacgcugucggugag.................................................................   918       0         seq
    .......................aacauucaacgcugucggugagu................................................................   9280      0         seq
    .......................aacauucaacgcugucggugaguu...............................................................   260       0         seq
    .......................aacauucaacgcugucggugaguuu..............................................................   127       0         seq
    ........................acauucaacgcugucggu....................................................................   1         0         seq
    ........................acauucaacgcugucggug...................................................................   1         0         seq
    ........................acauucaacgcugucgguga..................................................................   4         0         seq
    ........................acauucaacgcugucggugagu................................................................   14        0         seq
    .........................cauucaacgcugucggugag.................................................................   1         0         seq
    .............................................................aaaccaucgaccguugauugu............................   1         0         seq
    ..............................................................aaccaucgaccguugauuguacc.........................   2         0         seq
    ...............................................................accaucgaccguugauugu............................   12        0         seq
    ...............................................................accaucgaccguugauugua...........................   1         0         seq
    ...............................................................accaucgaccguugauuguac..........................   9         0         seq
    ...............................................................accaucgaccguugauuguacc.........................   26        0         seq
```
